# Lumpy skin disease virus LSDV001 protein positively regulates inflammatory response by promoting assembly of the TAK1-TAB2/3 complex

**DOI:** 10.1128/mbio.01677-25

**Published:** 2025-08-25

**Authors:** Yu-Lin Yang, Huai-Jie Jia, Meng-Yao Sun, Ai-Min Guo, Tian Xia, Hong-Bing Shu, Li-Bo Cao

**Affiliations:** 1 State Key Laboratory for Animal Disease Control and Prevention, Lanzhou Veterinary Research Institute, Chinese Academy of Agricultural Sciences; College of Veterinary Medicine, Lanzhou University; Gansu Province Research Center for Basic Disciplines of Pathogen Biology 111658 , Lanzhou, China; 2 Department of Infectious Diseases, Medical Research Institute, Taikang Center for Life and Medical Sciences, Zhongnan Hospital of Wuhan University, Wuhan University 89674, Wuhan, China; Huazhong Agricultural University, Wuhan, Hubei, China

**Keywords:** lumpy skin disease virus, inflammation, pathogenesis, LSDV001, TAK1-TAB2/3

## Abstract

Lumpy skin disease (LSD) is an emerging transboundary viral disease of livestock caused by the lumpy skin disease virus (LSDV), which has caused substantial economic losses and negatively impacted veterinary public health. The underlying pathogenic mechanisms remain poorly understood, limiting the effective prevention and control of LSD. Here, we identify LSDV-encoded protein LSDV001 as a positive regulator of IL-1β- and TNFα-triggered signaling. LSDV001 interacts with TAK1 and TAB2/3 and promotes assembly of the TAK1-TAB2/3 complex. This leads to IKK-dependent activation of the transcription factor NF-κB and induction of downstream inflammatory cytokines. LSDV001-deficient virus (LSDVΔ001) has attenuated the ability to activate NF-κB and induce the expression of inflammatory cytokines. Infection with LSDVΔ001 leads to smaller skin nodules and reduced inflammation compared to wild-type LSDV. Our findings suggest that LSDV001 acts as a key virulence factor of LSDV by promoting excessive inflammatory response upon infection.

**IMPORTANCE:**

Lumpy skin disease is a current global concern caused by the lumpy skin disease virus (LSDV), for which there is a lack of safe and efficient vaccines. In this study, we report that LSDV001 protein potentiates IL-1β- and TNFα-triggered IKK-dependent activation of NF-κB and transcription of inflammatory cytokines. Mechanistically, LSDV001 enhances inflammatory response by interacting with TAK1 and TAB2/3 to promote TAK1-TAB2/3 complex formation. We further demonstrate that LSDV001 deficiency attenuates LSDV-triggered inflammatory response and pathogenesis. Our findings identify a new virulence factor and reveal a novel pathogenic mechanism of LSDV by which LSDV001 enhances inflammatory response.

## INTRODUCTION

Lumpy skin disease (LSD) is an emerging transboundary viral disease caused by the lumpy skin disease virus (LSDV) that infects large domesticated ruminants such as cattle, water buffalo, and wild bovine species (1, 2). In recent years, LSD has rapidly spread around the world, resulting in reduced milk production, damage to hides, sterility, abortions, weight loss, and even death of cattle (3, 4). The World Organization for Animal Health (WOAH) has listed LSD as one of the most economically important and notifiable transboundary viral animal diseases (5). Typical clinical manifestations of LSD include fever, loss of appetite, and nodular lesions covering the whole body, mucosa, and organs (3, 6). Histopathological observations of nodular lesions have revealed hyperplasia and ballooning degeneration, along with the accumulation of inflammatory exudates in the cellular layers of both epidermis and dermis (7–9). It has been reported that LSDV-infected cattle exhibit marked infiltration of inflammatory cells at the infection sites and significant elevation of inflammatory cytokines in the serum compared to healthy cattle. The imbalance of the inflammatory response prolongs the chronic infection and extends negative consequences for animal health (1, 10). Therefore, investigating the mechanisms of LSDV-induced inflammatory imbalance may reveal the intricate host-virus dynamics during disease progression and provide valuable insights and direction for the prevention and control of LSD.

The proinflammatory cytokines IL-1β and TNFα are central regulators in the initiation of inflammatory response, which also play critical roles in the pathogenesis of inflammatory diseases (11, 12). IL-1β- and TNFα-triggered signaling pathways have been intensively studied. The binding of IL-1β to its receptor, IL-1R, initiates the recruitment of the IL-1R accessory protein (IL-1RAcP) and the adaptor protein MyD88 (13, 14). MyD88 further recruits IRAK1 and IRAK4 to the receptor-associated complex, in which IRAK4 phosphorylates IRAK1, resulting in the release of IRAK1 from the complex. The released IRAK1 associates with the E3 ligase TRAF6 to form a signalosome, in which TRAF6 catalyzes its autoubiquitination and the synthesis of unanchored K63-linked polyubiquitin chains. The TRAF6-associated K63-linked polyubiquitin chains preferentially bind to TAB2 and TAB3, which then recruit TAK1-TAB1 to the complex, leading to oligomerization and autophosphorylation of TAK1 (15–21). The activated TAK1 further activates IKKβ, leading to the phosphorylation and degradation of IκBα and activation of the transcription factor NF-κB (22–24).

Upon binding to TNFα, TNF-R1 recruits the adaptor protein TRADD via their respective death domains. TRADD then recruits cIAP1/2, TRAF2, and RIP1 to form a large receptor complex, where TRAF2 catalyzes K63-linked polyubiquitination of RIP1 (25–27). In the TNF-R1 complex, TRAF2 can recruit TAB2/3, which further recruits TAK1-TAB1 to the complex, leading to IKKβ-dependent NF-κB activation. Alternatively, RIP-linked polyubiquitin chains recruit NEMO/IKKγ to directly activate the IKK complex (21, 28–30). The activated NF-κB induces transcription of a large set of downstream effector genes, particularly inflammatory cytokines such as TNFα, IL-1β, IL-6, and various chemokines, leading to inflammatory response and host defense (31). However, excessive inflammatory response causes detrimental local or systemic inflammatory reactions and severe diseases.

In this study, we identified the LSDV-encoded LSDV001 protein as a positive regulator of IL-1β- and TNFα-triggered signaling. Our results indicated that LSDV001 promoted assembly of the TAK1-TAB2/3 complex, thereby facilitating NF-κB activation and induction of inflammatory genes. LSDV001-deficient virus (LSDVΔ001) exhibited attenuated ability to induce inflammatory cytokines and formation of skin nodules compared with wild-type LSDV. Our findings suggest that LSDV001 is a virulence factor of LSDV that contributes to the excessive inflammatory response and pathogenesis induced by LSDV infection.

## RESULTS

### LSDV infection induces proinflammatory cytokines and chemokines

**Fig 1:**
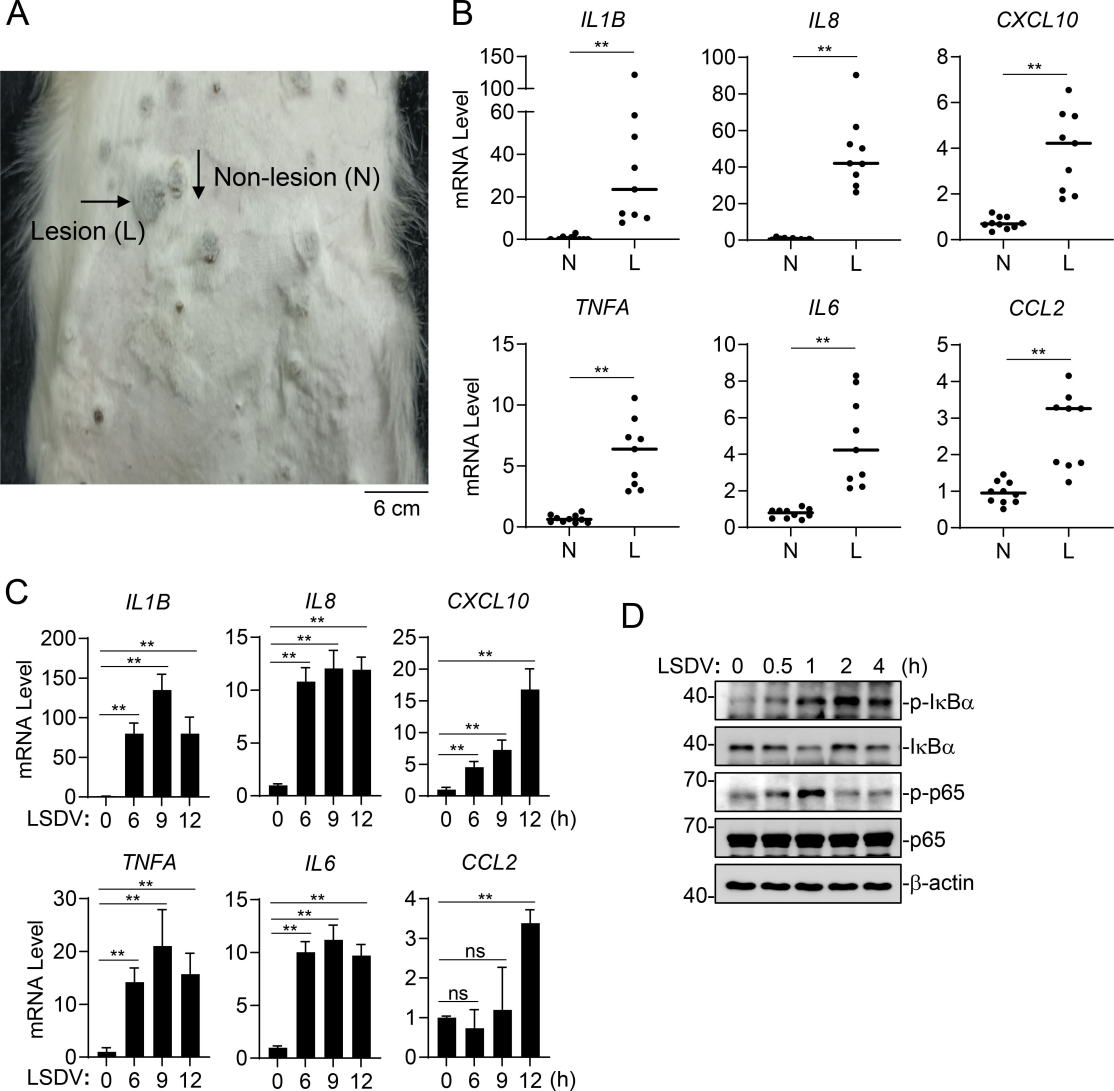
LSDV infection induces proinflammatory cytokines and chemokines. (**A and B**) LSDV induces the transcription of inflammatory cytokines and chemokines *in vivo*. Holstein cattle were intravenously injected with LSDV for 14 days, and then lesion (**L**) and non-lesion (**N**) skin samples from the same cattle were dissected for qPCR analysis of mRNA levels of the indicated inflammatory cytokine and chemokine genes. (**C**) LSDV induces the transcription of inflammatory cytokines and chemokines in MDBK cells. MDBK cells were left uninfected or infected with LSDV for the indicated times, followed by qPCR analysis of mRNA levels of the indicated inflammatory cytokine and chemokine genes. (**D**) LSDV induces activation of p65 and IκBα in MDBK cells. MDBK cells were left uninfected or infected with LSDV for the indicated times before immunoblot analysis. Data are the mean ± SD (*n* = 9 for lesion skin group and *n* = 10 for non-lesion skin group in A and B, or *n* = 3 in C) from one representative experiment. ns, nonsignificant; **P < 0.01 (unpaired t-test).

Previous studies have shown that imbalanced inflammatory response and the production of excessive inflammatory cytokines lead to long-term nodule formation and organ inflammation in LSDV-infected cattle (1, 10). NF-κB activation is critical for the inducible expression of the inflammatory cytokines such as IL-1β, IL-6, IL-8, and TNFα. Both IL-1β and TNFα further activate NF-κB to initiate autoregulatory pathways that are thought to be responsible for increasing the magnitude of the inflammatory response (32–34). To delineate the molecular basis for the imbalanced inflammatory response, we first examined the activation of inflammatory signaling pathways during LSDV infection. We established an LSDV infection model *in vivo* and dissected lesion and non-lesion skin samples from the same Holstein cattle for RT-qPCR. The results indicated that the transcription of inflammatory cytokines and chemokines, including *IL-1β*, *IL6*, *IL8*, *TNFA*, *CXCL10,* and *CCL2*, was upregulated in skin lesions (Fig. 1A and B). Consistently, LSDV markedly induced the transcription of these inflammatory cytokines and chemokines in Madin-Darby bovine kidney (MDBK) cells (Fig. 1C). Immunoblot analysis further showed that LSDV induced the phosphorylation of IκBα and p65, which are hallmarks for the activation of signaling triggered by the proinflammatory cytokines IL-1β and TNFα (Fig. 1D). These results suggest that LSDV infection induces proinflammatory cytokines and chemokines.

### LSDV001 positively regulates IL-1β- and TNFα-triggered signaling

**Fig 2:**
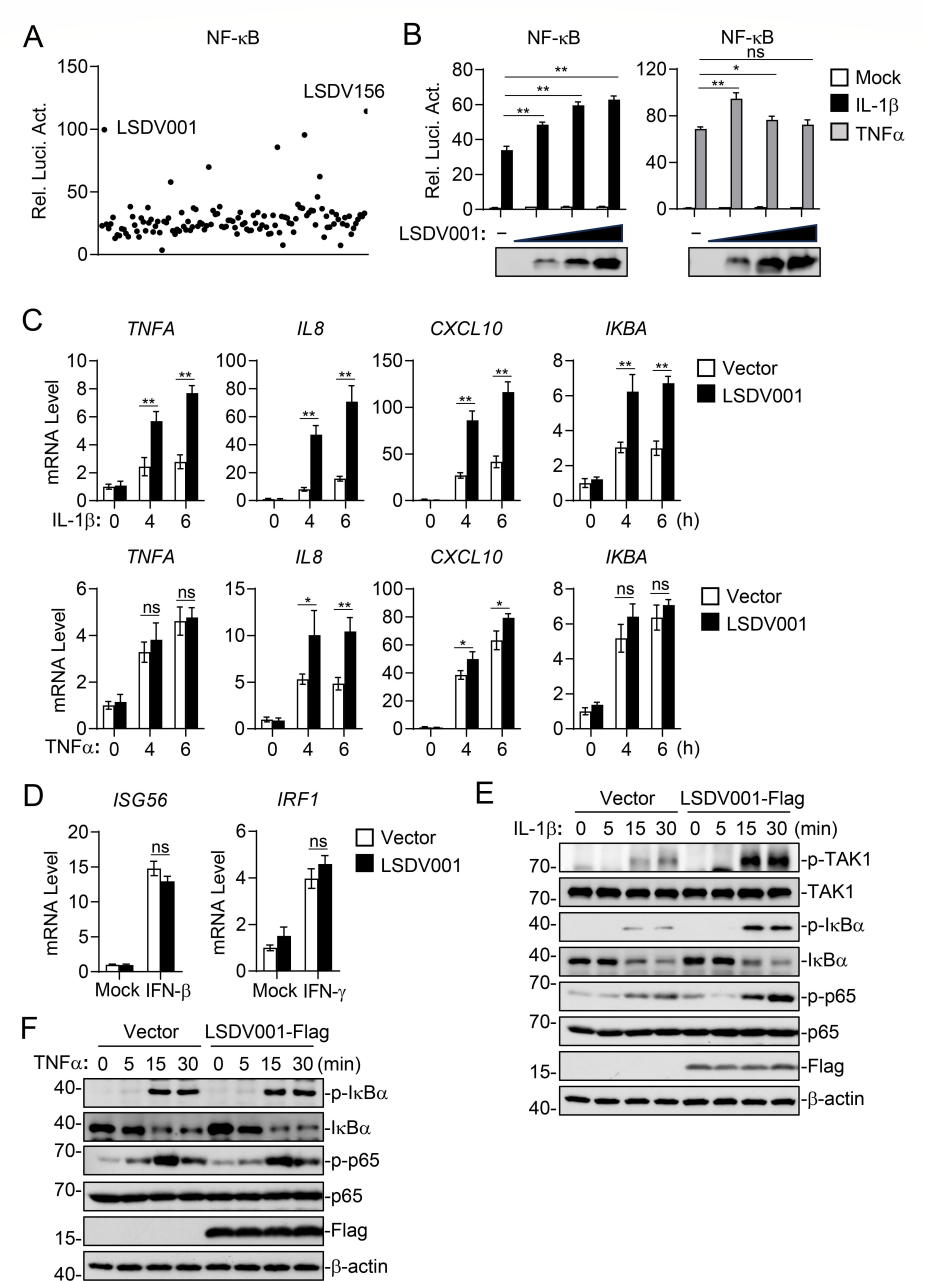
LSDV001 positively regulates IL-1β-triggered signaling and has minimal effects on TNFα-triggered signaling in HEK293 cells. (**A**) Screening of LSDV proteins that regulate activation of NF-κB induced by IL-1β. HEK293 cells (1 × 10^5^) were transfected with pRL-TK (20 ng), NF-κB reporter (20 ng), and LSDV eukaryotic expression plasmids (200 ng) for 24 hours. The cells were then treated with IL-1β or left untreated for 10 hours before reporter assays. (**B**) Effects of LSDV001 on IL-1β and TNFα-induced NF-κB activation. HEK293 cells (1 × 10^5^) were transfected with pRL-TK (20 ng), NF-κB reporter (20 ng), and LSDV001 plasmid (1, 10, and 20 ng) for 24 hours. The cells were then treated with IL-1β, TNFα, or left untreated for 10 hours before reporter assays. (**C**) Effects of LSDV001 on IL-1β and TNFα-induced transcription of inflammatory cytokines. HEK293 cells (5 × 10^5^) were transfected with either an empty vector or LSDV001-Flag plasmid for 24 hours. The cells were then treated with IL-1β, TNFα, or left untreated for the indicated times before qPCR experiments. (**D**) Effects of LSDV001 on IFN-β and IFN-γ-induced transcription of downstream genes. HEK293 cells (5 × 10^5^) were transfected with either an empty vector or LSDV001-Flag plasmid for 24 hours. The cells were then treated with IFN-β, IFN-γ, or left untreated for 6 hours before qPCR experiments. (**E-F**). Effects of LSDV001 on IL-1β and TNFα-induced phosphorylation of signaling molecules. HEK293 cells (5 × 10^5^) were transfected with either an empty vector or LSDV001-Flag plasmid for 24 hours. The cells were then treated with IL-1β, TNFα, or left untreated for the indicated times before immunoblot analysis. Data shown in B–D are mean ± SD (*n* = 3) from one representative experiment. ns, nonsignificant; *P < 0.05; **P < 0.01 (unpaired t-test).

We next investigated whether LSDV-encoded proteins regulate the inflammatory response. We screened 156 LSDV proteins for their abilities to regulate IL-1β-induced activation of NF-κB by reporter assays in human embryonic kidney 293 (HEK293) cells. These screens led to the identification of LSDV001 and LSDV156, which markedly enhanced IL-1β-induced NF-κB activation (Fig. 2A). LSDV001 and LSDV156 are identical proteins encoded by two reversely repeated genes, *LSDV001* and *LSDV156*. For simplification, we referred to this protein as LSDV001. Overexpression of LSDV001 potentiated IL-1β-induced NF-κB activation in a dose-dependent manner in HEK293 cells. In similar experiments, LSDV001 enhanced TNFα-induced activation of NF-κB at low doses, whereas it lost its effects at higher doses in HEK293 cells (Fig. 2B). In addition, overexpression of LSDV001 promoted IL-1β-induced transcription of *TNFA*, *IL8*, *CXCL10,* and *IKBA* genes in HEK293 cells (Fig. 2C). Overexpression of LSDV001 promoted TNFα-induced transcription of *IL8* and *CXCL10* genes to lower degrees and had no marked effects on TNFα-induced transcription of *TNFA* and *IKBA* genes in HEK293 cells (Fig. 2C). In similar experiments, overexpression of LSDV001 had no marked effects on IFN-β-induced transcription of *ISG56* or IFN-γ-induced transcription of *IRF1* gene in HEK293 cells (Fig. 2D). Biochemically, overexpression of LSDV001 enhanced IL-1β-induced phosphorylation of TAK1, IκBα, and p65, which are hallmarks of activation of the NF-κB pathways (Fig. 2E), whereas LSDV001 had no marked effects on TNFα-induced phosphorylation of IκBα and p65 in HEK293 cells (Fig. 2F). These results suggest that LSDV001 positively regulates IL-1β-triggered signaling but has minimal effects on TNFα-triggered signaling in HEK293 cells.

**Fig 3:**
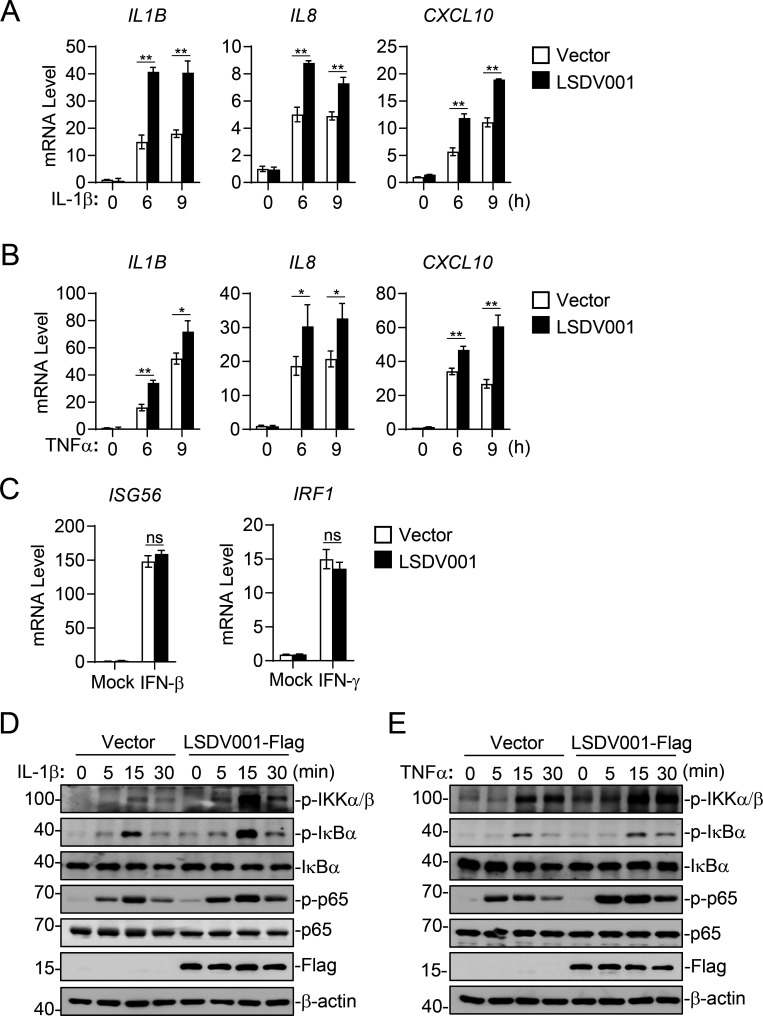
LSDV001 positively regulates IL-1β- and TNFα-triggered signaling in MDBK cells. (**A and B**). Effects of LSDV001 on IL-1β and TNFα-induced transcription of inflammatory cytokines. MDBK cells (2 × 10^5^) were transduced with either an empty vector or LSDV001-Flag plasmid to establish stable cell lines. The cells (2 × 10^5^) were treated with IL-1β, TNFα, or left untreated for the indicated times before qPCR experiments. (**C**) Effects of LSDV001 on IFN-β and IFN-γ-induced transcription of downstream genes. MDBK cells (2 × 10^5^) stably transduced with an empty vector or LSDV001-Flag plasmid were treated with IFN-β, IFN-γ, or left untreated for 6 hours before qPCR experiments. (**D and E**). Effects of LSDV001 on IL-1β and TNFα-induced phosphorylation of IKKα/β, IκBα, and p65. MDBK cells (2 × 10^5^) stably transduced with an empty vector or LSDV001-Flag plasmid were treated with IL-1β, TNFα, or left untreated for the indicated times before immunoblot analysis. Data shown in A–C are mean ± SD (*n* = 3) from one representative experiment. ns nonsignificant; *P < 0.05, **P < 0.01 (unpaired t-test).

Since LSDV is a pathogen of ruminants but not humans, we next investigated the effects of LSDV001 on IL-1β and TNFα signaling in Madin-Darby bovine kidney (MDBK) cells. We first established an MDBK-LSDV001 stable cell line ectopically expressing C-terminal Flag-epitope tagged LSDV001. RT-qPCR experiments indicated that ectopic expression of LSDV001 potentiated both IL-1β- and TNFα-induced transcription of *IL1B*, *IL8,* and *CXCL8* genes in MDBK cells (Fig. 3A and B). In similar experiments, ectopic expression of LSDV001 did not affect IFN-β-induced transcription of *ISG56* or IFN-γ-induced transcription of *IRF1* gene in MDBK cells (Fig. 3C). Immunoblotting analysis indicated that LSDV001 enhanced both IL-1β- and TNFα-induced phosphorylation of IKKα/β, IκBα, and p65 in MDBK cells (Fig. 3D and E). These results suggest that LSDV001 potentiates IL-1β- and TNFα-triggered signaling and induction of inflammatory genes in MDBK cells.

### LSDV001 interacts with TAK1 and TAB2/3

**Fig 4:**
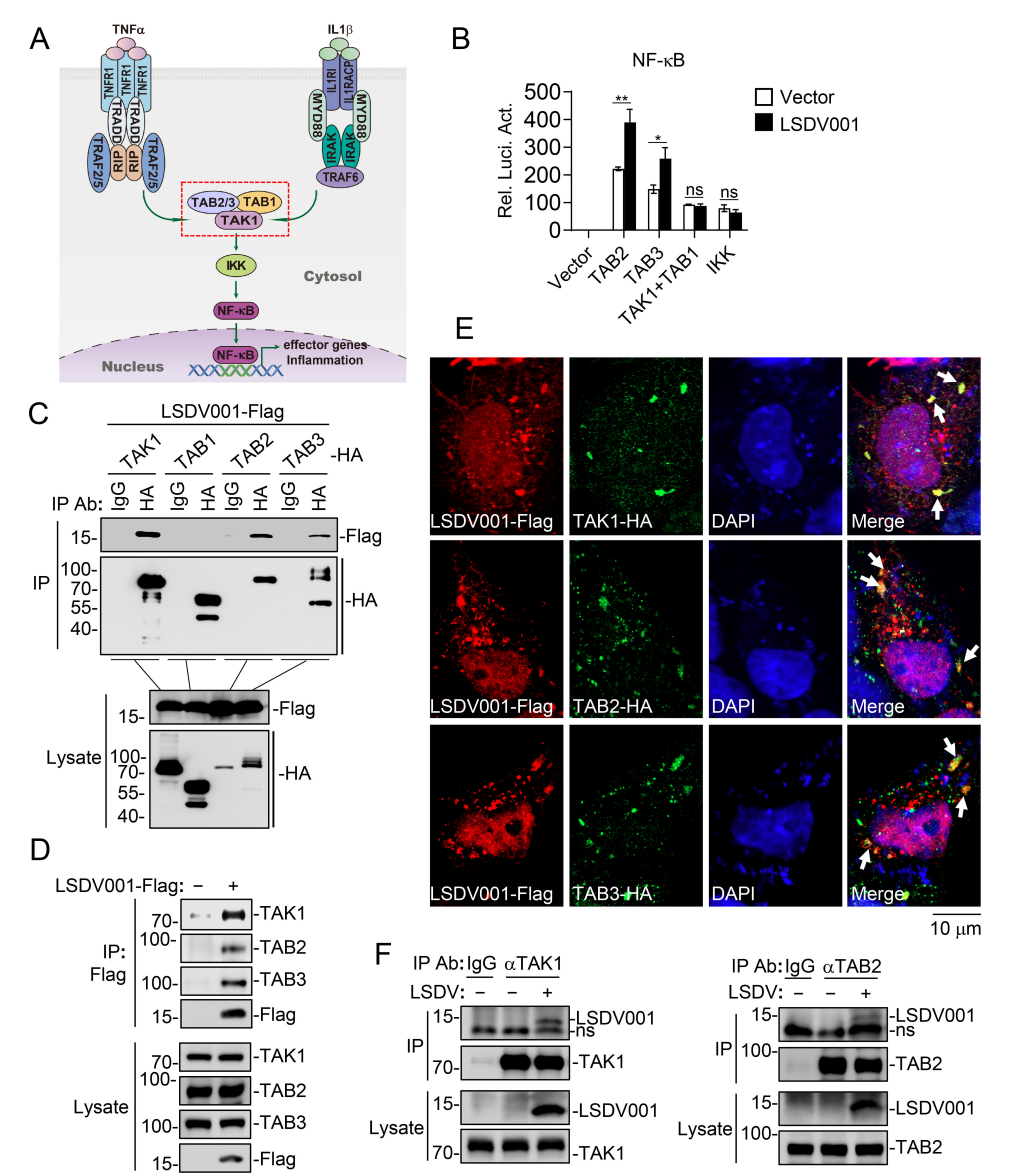
LSDV001 interacts with TAK1 and TAB2/3. (**A**) Schematic diagram of IL-1β and TNFα signaling. (**B**) Effects of LSDV001 on NF-κB activation mediated by various components. HEK293 cells (1 × 10^5^) were transfected with pRL-TK (20 ng), NF-κB reporter (20 ng), and indicated plasmids for 24 hours before reporter assays. Data shown are mean ± SD (*n* = 3) from one representative experiment. ns, nonsignificant; *P < 0.05, **P < 0.01 (unpaired t-test). (**C**) The interaction of LSDV001 with TAK1 and TABs in the overexpression system. HEK293 cells (1 × 10^7^) were transfected with the indicated plasmids for 24 hours and then lysed for coimmunoprecipitation with control IgG or HA antibody, followed by immunoblot analysis with the indicated antibodies. (**D**) The interaction of LSDV001 with endogenous TAK1 and TAB2/3. HEK293 cells (2 × 10^7^) were transfected with an empty vector or LSDV001-Flag plasmid for 24 hours and then lysed for coimmunoprecipitation with Flag antibody, followed by immunoblot analysis with the indicated antibodies. (**E**) Co-localization of LSDV001 with TAK1 and TAB2/3. HeLa cells (2 × 10^4^) were transfected with the indicated plasmids for 20 hours and then fixed for immunostaining before being subjected to confocal microscopy. (**F**) The interaction of endogenous LSDV001 with TAK1 and TAB2 during LSDV infection. MDBK cells (5 × 10^7^) were left uninfected or infected with LSDV (MOI = 2) before cells were harvested for immunoprecipitation with control IgG or antibodies against TAK1 or TAB2. The lysates and immunoprecipitates were subjected to immunoblot analysis with the indicated antibodies.

Since IL-1β and TNFα signaling pathways converge at the TAK1-TAB2/3 complex (29, 31), and our results indicated that overexpression of LSDV001 promoted phosphorylation of TAK1 and its downstream components IKKβ, IκBα, and p65, we hypothesized that LSDV001 functioned at the level of the TAK1-TAB2/3 complex (Fig. 4A). To test this, we examined the effects of LSDV001 on NF-κB activation by components of the complex by reporter assays. The results indicated that LSDV001 potentiated NF-κB activation mediated by overexpression of TAB2 or TAB3 but had no marked effects on overexpression of TAK1 and TAB1, which constitutively associate with each other in unstimulated cells (35). Overexpression of LSDV001 also did not promote IKKβ-mediated NF-κB activation in reporter assays (Fig. 4B). Co-immunoprecipitation experiments indicated that LSDV001 interacted with TAK1, TAB2, and TAB3 but not TAB1 (Fig. 4C). Furthermore, we found that LSDV001 interacted with endogenous TAK1 and TAB2/3 (Fig. 4D). Confocal microscopy also indicated that LSDV001 colocalized with TAK1 and TAB2/3 (Fig. 4E). We further evaluated the interactions of LSDV001 with TAK1 and TAB2 during LSDV infection. Co-immunoprecipitation experiments showed that LSDV001 was associated with endogenous TAK1 and TAB2 in LSDV-infected MDBK cells (Fig. 4F). These results suggest that LSDV001 targets TAK1 and TAB2/3 to promote inflammatory response.

**Fig 5:**
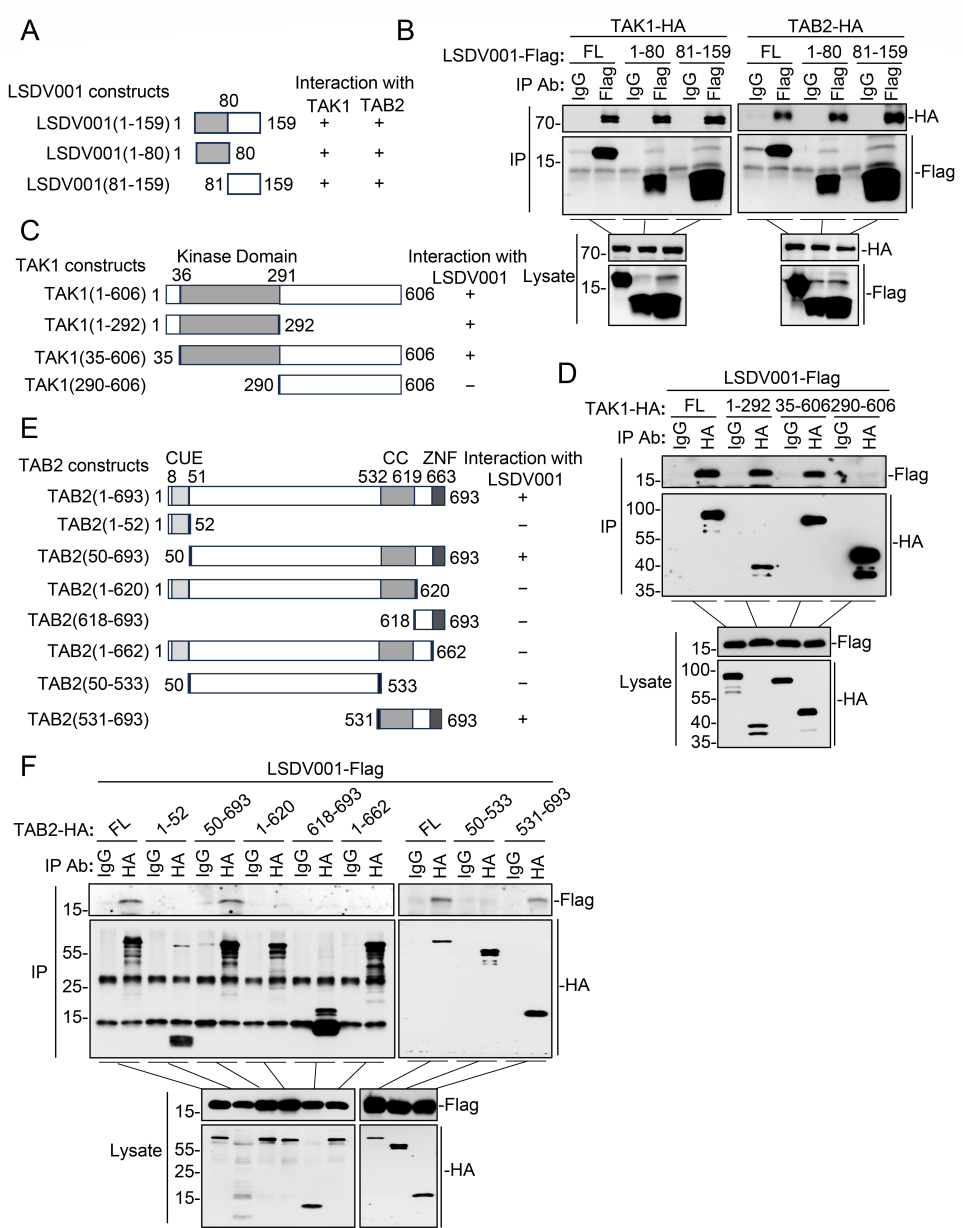
Domain mapping of the interactions between LSDV001 and TAK1 or TAB2. (**A**) A schematic presentation of LSDV001 truncations and their abilities to interact with TAK1 and TAB2. (**B**) The interaction of LSDV001 truncations with TAK1 and TAB2. HEK293 cells (1 × 10^7^) were transfected with the indicated plasmids for 24 hours, followed by coimmunoprecipitation and immunoblot analysis. (**C**) Schematic presentation of TAK1 truncations and their abilities to interact with LSDV001. (**D**) The interaction of TAK1 truncations with LSDV001. HEK293 cells (1 × 10^7^) were transfected with the indicated plasmids for 24 hours, followed by coimmunoprecipitation and immunoblot analysis. (**E**) A schematic presentation of TAB2 truncations and their abilities to interact with LSDV001. (**F**) The interaction of TAB2 truncations with LSDV001. HEK293 cells (1 × 10^7^) were transfected with the indicated plasmids for 24 hours, followed by coimmunoprecipitation and immunoblot analysis. −, no interaction; +, positive interaction.

We next mapped the domains responsible for the interaction of LSDV001 with TAK1 and TAB2/3. Since LSDV001 is a 159 aa protein without a recognizable domain, we arbitrarily made two truncation mutants, which contain either the N-terminal 80 aa (LSDV001-N) or the C-terminal 79 aa (LSDV001-C). The truncation constructs of TAK1 and TAB2 were designed based on their respective functional domains reported in previous studies (36, 37). Coimmunoprecipitation experiments indicated that both LSDV001-N and LSDV001-C interacted with TAK1 and TAB2 (Fig. 5A and B). We also made a series of truncations of TAK1 and TAB2 and performed transient transfection and coimmunoprecipitation experiments to detect their interaction with LSDV001. The results indicated that the N-terminal kinase domain of TAK1 was sufficient for its association with LSDV001 (Fig. 5C and D). The coiled coil (CC) and Npl4 zinc finger (NZF) domains (532–693 aa) of TAB2 were sufficient for its interaction with LSDV001 (Fig. 5E and F). Since TAB2 and TAB3 are highly homologous and have redundant functions (38), it would be expected that LSDV001 interacts with the CC and NZF domains of TAB3. Previously, it has been shown that the NZF domains of TAB2/3 bind to the K63-linked polyubiquitin chains associated with TRAF6 (19), and the C-terminal CC-containing domain of TAK1 associates with the C-terminus of TAB2/3 after IL-1β stimulation (18, 39). In light of these observations, LSDV001 may act as an additional linker by its simultaneous interaction with the CC-NZF domains of TAB2/3 and the N-terminal kinase domain of TAK1 following LSDV infection.

### LSDV001 promotes assembly of the TAK1-TAB2/3 complex

**Fig 6:**
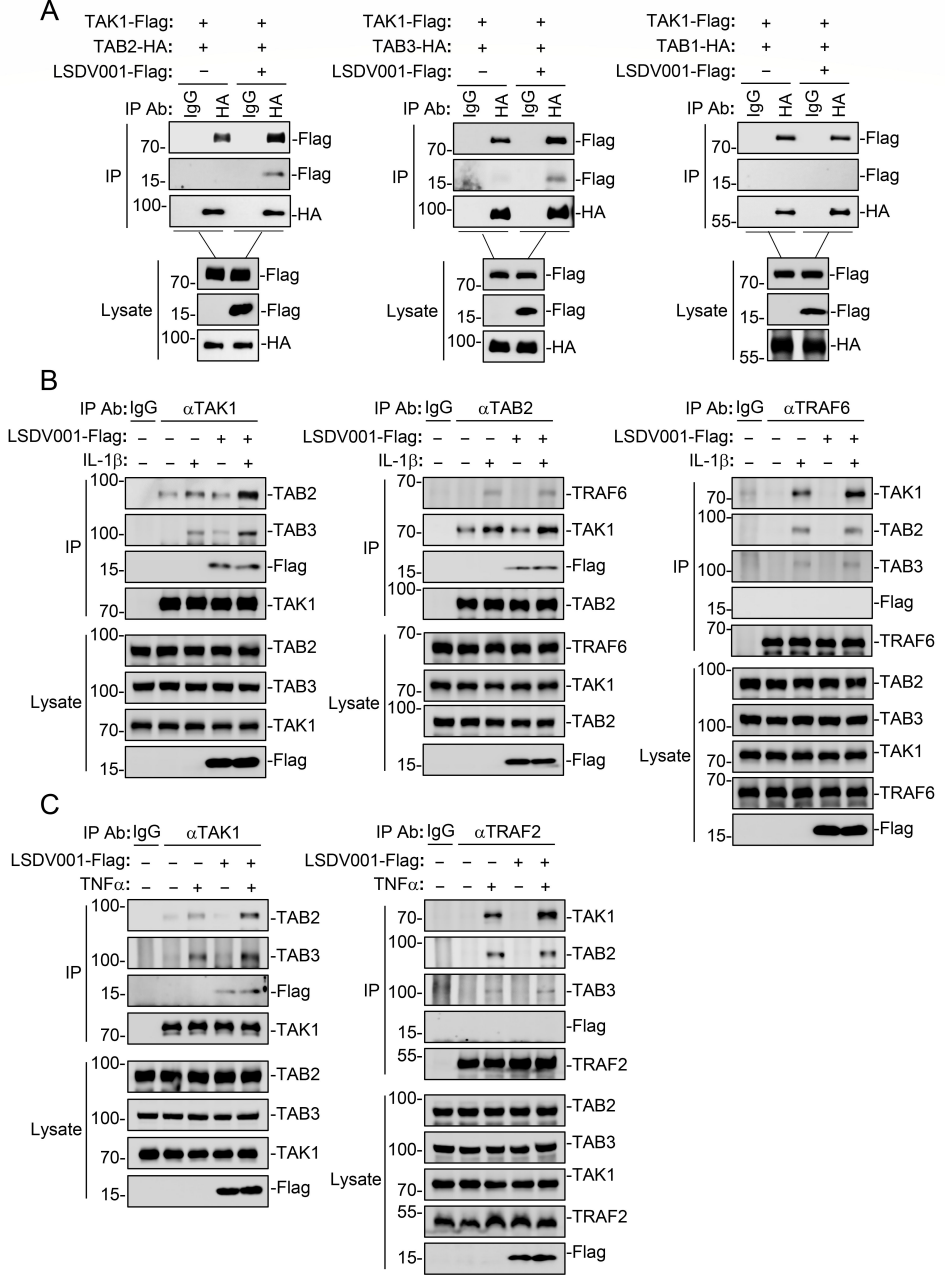
LSDV001 promotes assembly of the TAK1-TAB2/3 complex. (**A**) Effects of LSDV001 on the association of TAK1 with TABs. HEK293 cells (1 × 10^7^) were transfected with the indicated plasmids for 24 hours, followed by coimmunoprecipitation and immunoblot analysis. (**B and C**) Effects of LSDV001 on the endogenous association of TRAF6 or TRAF2 with TAK1 and TAB2/3, as well as TAB2/3 with TAK1 following IL-1β or TNFα stimulation. HEK293 cells (2 × 10^7^) were transfected with an empty vector or LSDV001-Flag plasmid for 24 hours. The cells were left untreated or treated with IL-1β or TNFα for 15 minutes before cells were harvested for immunoprecipitation with control IgG or antibodies against TAK1, TAB2, TRAF6, or TRAF2. The lysates and immunoprecipitates were subjected to immunoblot analysis with the indicated antibodies.

It has been demonstrated that assembly of the TAK1-TAB2/3 complex is essential for IL-1β and TNFα signaling (40). We next determined whether LSDV001 promotes assembly of the TAK1-TAB2/3 complex. Co-immunoprecipitation assays showed that LSDV001 enhanced the interaction of TAK1 with TAB2 or TAB3, whereas LSDV001 did not affect the association of TAK1 with TAB1 (Fig. 6A), which constitutively associate with each other in unstimulated cells (35). Ectopic expression of LSDV001 enhanced IL-1β-induced endogenous association of TRAF6 with TAK1, as well as TAB2/3 with TAK1, whereas LSDV001 had no marked effects on IL-1β-induced association between TRAF6 and TAB2/3 (Fig. 6B). Consistently, LSDV001 also enhanced TNFα-induced endogenous association of TRAF2 with TAK1 and TAB2/3 with TAK1, but not the association between TRAF2 and TAB2/3 (Fig. 6C). Previous studies have shown that TAB2/3 is preferentially recruited to signaling complexes such as IRAK1-TRAF6 or TRAF2-RIP1 following IL-1β or TNFα stimulation, where it subsequently facilitates the recruitment of TAK1 and the activation of downstream signaling (18, 19, 21). Combined with our results, we suggest that LSDV001 facilitates assembly of the TAK1-TAB2/3 complex, thereby enhancing the interaction of TRAF6 or TRAF2 with TAK1 and the activation of TAK1 and NF-κB following IL-1β or TNFα stimulation.

### LSDV001 deficiency attenuates LSDV-triggered inflammatory response and pathogenesis

**Fig 7:**
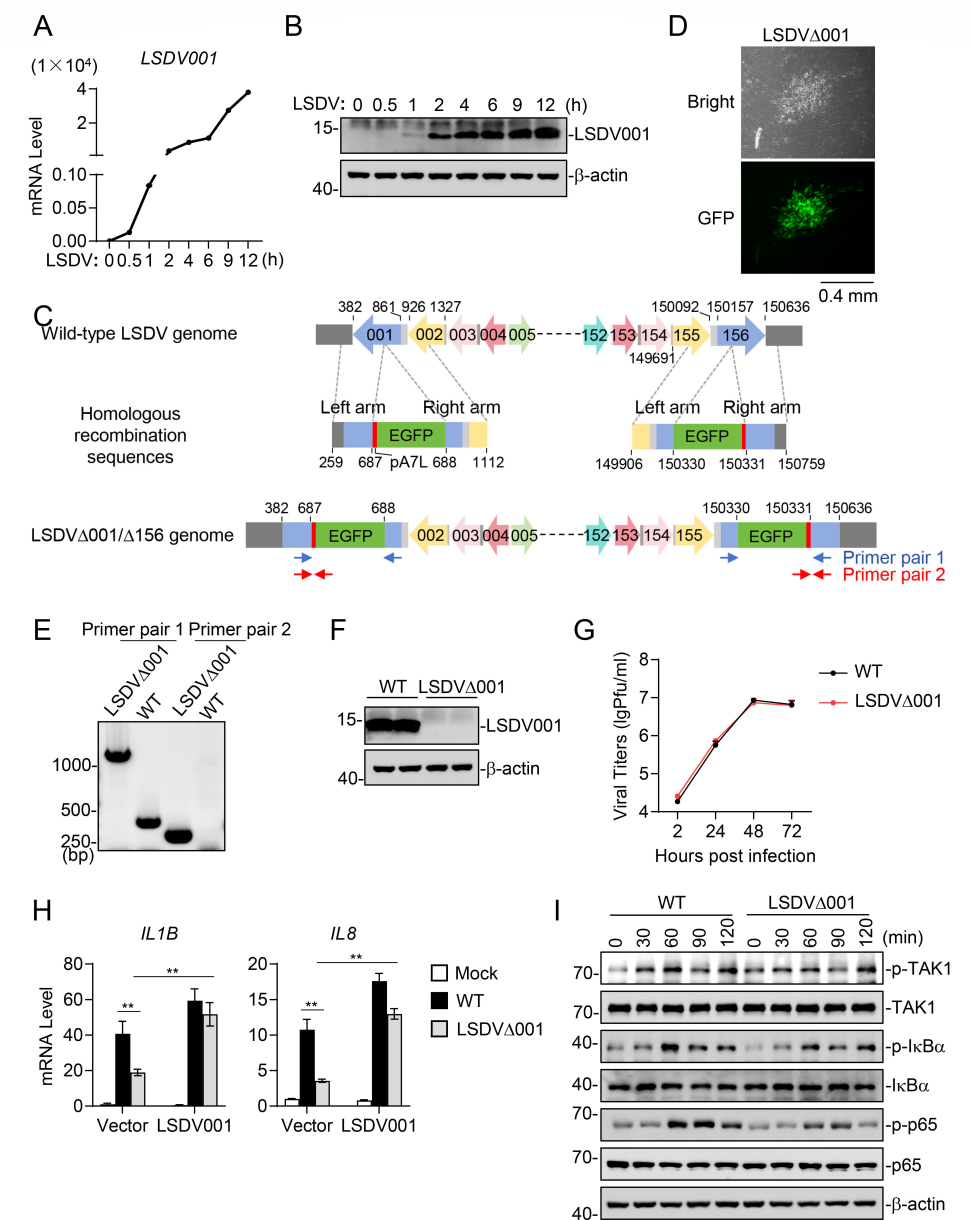
LSDV001 deficiency attenuates LSDV-triggered inflammatory response. (**A**) Kinetics of *LSDV001* gene transcription. MDBK cells were infected with LSDV (MOI = 2) for the indicated times, followed by qPCR analysis of mRNA levels of the *LSDV001* gene. (**B**) Kinetics of LSDV001 protein expression. MDBK cells were infected with LSDV (MOI = 2) for the indicated times before immunoblot analysis with an LSDV001 antibody. (**C**) Schematic diagram of the generation of LSDVΔ001/Δ156. LSDVΔ001/Δ156 was generated by homologous recombination in MDBK cells. The recombination cassette contained left and right homology arms flanking the *LSDV001* and *LSDV156* loci, as well as the *EGFP* reporter gene driven by the LSDV pA7L promoter, which was inserted into the coding region of *LSDV001* and *LSDV156*. Identical colors in the diagram represent identical nucleotide sequences. (**D**) EGFP expression of LSDVΔ001 was observed by fluorescence microscopy. (**E**) PCR verification of LSDVΔ001 purity. Virus DNA of wild-type LSDV or LSDVΔ001 (MOI = 2) was amplified with specific primers before gel electrophoresis analysis. (**F**) Immunoblot verification of LSDVΔ001 purity. MDBK cells were infected with wild-type LSDV or LSDVΔ001 (MOI = 2), and the cells were then lysed and expression of LSDV001 was detected by immunoblot analysis. (**G**) Growth kinetics of wild-type LSDV and LSDVΔ001. MDBK cells were infected with wild-type LSDV or LSDVΔ001 (MOI = 0.01) for the indicated times before plaque assays. (**H**) Effects of LSDV001 deficiency and restoration on LSDV-induced transcription of inflammatory cytokine and chemokine genes. MDBK control cells or MDBK cells stably expressing LSDV001 were left uninfected or infected with wild-type LSDV or LSDVΔ001 (MOI = 1) for the indicated times, followed by qPCR analysis of mRNA levels of the indicated genes. Data shown are mean ± SD (*n* = 3) from one representative experiment. **P < 0.01 (unpaired t-test). (**I**) Effects of LSDV001 deficiency on LSDV-induced phosphorylation of TAK1, IκBα, and p65. MDBK cells were left uninfected or infected with wild-type LSDV or LSDVΔ001 (MOI = 1) for the indicated times before immunoblot analysis with the indicated antibodies.

We next investigated the roles of LSDV001 in LSDV-triggered inflammatory response and pathogenesis. We first examined the kinetics of *LSDV001* transcription following LSDV infection. RT-qPCR experiments indicated that *LSDV001* mRNA was detected at 0.5 hours post-infection (Fig. 7A). Immunoblotting analysis also suggested that *LSDV001* was an early gene, as LSDV001 protein was detected at 1 hour after LSDV infection (Fig. 7B). To evaluate the function of endogenous LSDV001 on LSDV-triggered inflammatory response in MDBK cells, we generated a recombinant virus lacking LSDV001 (LSDVΔ001) from the highly pathogenic LSDV/China/Hainan/2021 by disrupting the *LSDV001* and *LSDV156* coding regions with LSDV A7L promoter-driven EGFP (Fig. 7C). The recombinant LSDVΔ001 viruses were purified by selecting EGFP-positive clones (Fig. 7D). The purity of LSDVΔ001 was confirmed by PCR analysis of the viral genome (Fig. 7E), and the deficiency of LSDV001 in LSDVΔ001 was verified by immunoblotting analysis of LSDV001 expression in cells infected with wild-type LSDV and LSDVΔ001 (Fig. 7F). We next compared the replication kinetics of LSDVΔ001 with that of wild-type LSDV in MDBK cells. The results indicated that wild-type LSDV and LSDVΔ001 replicated with similar kinetics (Fig. 7G), suggesting that LSDV001 does not play a role in LSDV replication. However, RT-qPCR experiments indicated that LSDVΔ001 induced markedly lower transcription levels of *IL1B* and *IL8* genes compared to wild-type LSDV in MDBK cells. The reduced transcription of *IL1B* and *IL8* genes following LSDVΔ001 infection was rescued in MDBK cells stably expressing LSDV001, where transcription of these genes was comparable to that induced by wild-type LSDV (Fig. 7H). Consistently, immunoblots indicated that phosphorylation of TAK1, IκBα, and p65 induced by LSDVΔ001 was markedly reduced compared to wild-type LSDV in MDBK cells (Fig. 7I). These results suggest that LSDV001 plays a critical role in LSDV-triggered inflammatory response.

**Fig 8:**
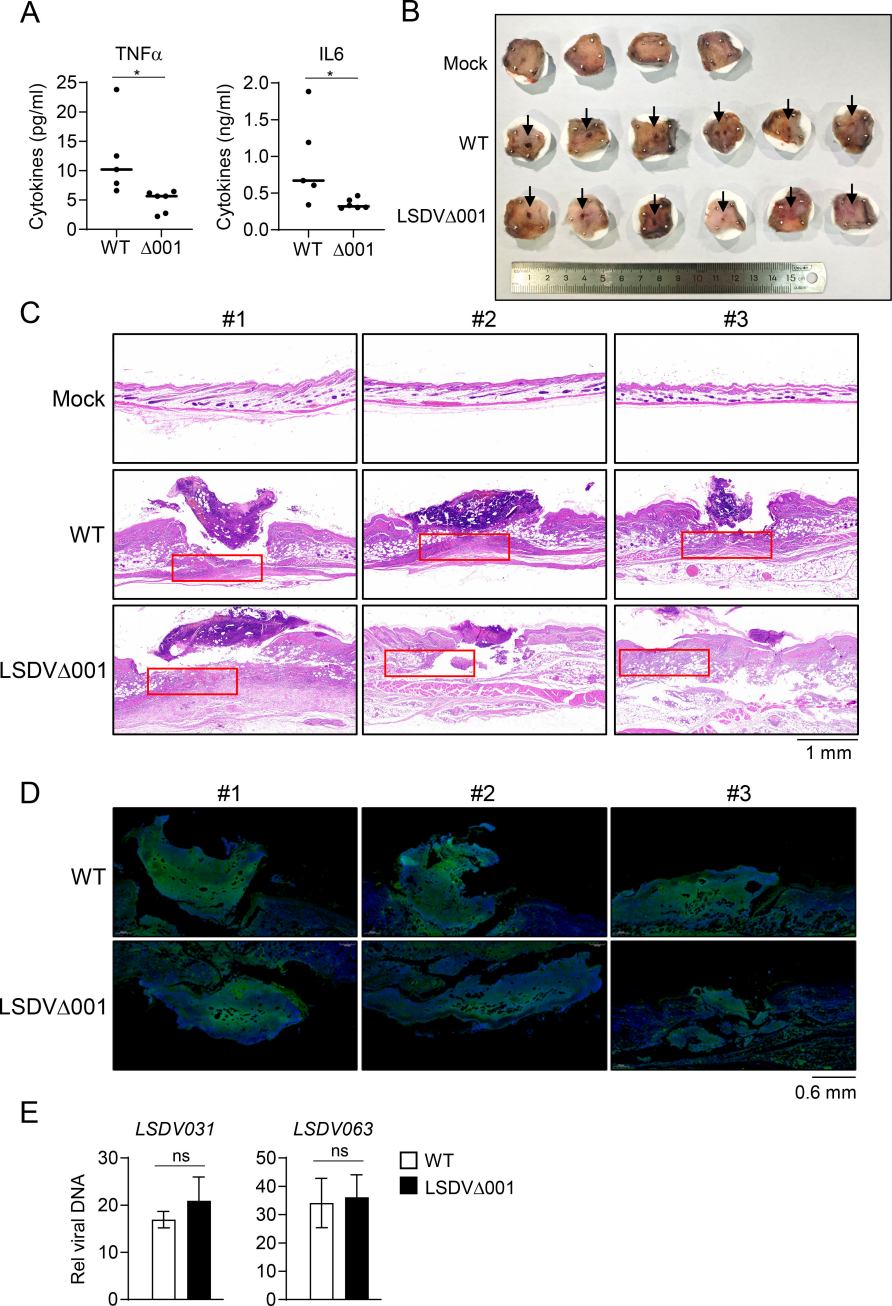
LSDV001 deficiency attenuates LSDV-triggered inflammatory response and pathogenesis *in vivo*. (**A**) Effects of LSDV001 deficiency on LSDV-induced production of TNFα and IL-6 in serum. Seven-week-old C57BL/6 mice (*n* = 5 for wild-type LSDV001 infection group, *n* = 6 for LSDVΔ001 infection group) were intravenously injected with wild-type LSDV or LSDVΔ001 (1 × 10^6^ pfu) for 24 hours, followed by orbital blood collection for ELISA. (**B**) Effects of LSDV001 deficiency on LSDV-induced formation of skin nodules. Three-week-old LVG hamsters (*n* = 3) were intradermally injected with wild-type LSDV or LSDVΔ001 (5 × 10^5^ pfu) for 5 days, and then the skin nodules were fixed and photographed. (**C**) Effects of LSDV001 deficiency on LSDV-induced histopathological changes of skin nodules. LVG hamsters (*n* = 3) were intradermally injected with wild-type LSDV, LSDVΔ001 (5 × 10^5^ pfu), or PBS for 5 days, and then the skin nodular lesions were fixed and stained with HE to observe pathological characteristics. Three representative fields from PBS, wild-type LSDV- or LSDVΔ001-injected groups were shown. The red box showed the tissue damage of skin nodular lesions. Any diagonal lines present in the images are stitching artifacts generated by the whole-slide scanner software during the assembly of the panoramic image from multiple fields of view. (**D**) Effects of LSDV001 deficiency on the viral loads of the skin nodules. LVG hamsters (*n* = 3) were intradermally injected with wild-type LSDV or LSDVΔ001 (5 × 10^5^ pfu) for 5 days, and then the skin nodular lesions were fixed and stained with DAPI for microscopy analysis. Three representative fields from wild-type LSDV- or LSDVΔ001-injected group were shown. (**E**) Effects of LSDV001 deficiency on the viral loads of the skin nodules. LVG hamsters (*n* = 3) were intradermally injected with wild-type LSDV or LSDVΔ001 (5 × 10^5^ pfu) for 5 days, and then the skin nodular lesions were harvested. Total DNA was extracted using a commercial viral DNA extraction kit. Viral loads were quantified by qPCR targeting the viral LSDV031 and LSDV063 genes. Data shown in A (*n* = 5 for wild-type LSDV001 infection group, *n* = 6 for LSDVΔ001 infection group) and E (*n* = 3) are mean ± SD from one representative experiment. ns, nonsignificant; *P < 0.05 (unpaired t-test).

We further evaluated the effects of LSDV001 on LSDV-triggered inflammatory response and pathogenesis *in vivo*. We found that the serum levels of TNFα and IL6 in mice infected with LSDVΔ001 were significantly lower than those in mice infected with wild-type LSDV (Fig. 8A). The formation of skin nodules is a characteristic clinical sign of LSDV pathogenesis (6, 8). It has been reported that Syrian hamsters are susceptible to LSDV and can be used as an *in vivo* model for studying LSDV infection (41). To examine the role of the *LSDV001* gene in LSDV pathogenicity, Syrian hamsters were intradermally infected with wild-type LSDV or LSDVΔ001. Five days after challenge with the viruses, nodules at the inoculation sites on the hamster skin were observed, and the nodular lesions in LSDVΔ001-infected hamsters were markedly smaller than those in wild-type LSDV-infected hamsters (Fig. 8B). Histopathological analysis revealed severe skin nodular lesions in the epidermis and dermis of wild-type LSDV-infected hamsters, characterized by significant necrotic cell debris and inflammatory exudates. These pathological phenomena were less severe in the LSDVΔ001-infected hamsters (Fig. 8C). As visualized by EGFP, which is tagged in both wild-type LSDV and LSDVΔ001, the viral loads in the skin nodular lesions of LSDVΔ001-infected hamsters were comparable to those in the wild-type LSDV-infected group (Fig. 8D). To further quantify viral replication at the lesion sites, total DNA was extracted from hamster skin nodules, followed by qPCR targeting the viral LSDV031 and LSDV063 genes. Consistent with the fluorescence imaging results, qPCR analysis confirmed comparable viral loads between the two groups (Fig. 8E). Taken together, these results suggest that LSDVΔ001 induces less inflammation and reduced disease severity compared to wild-type LSDV.

## DISCUSSION

In recent years, LSDV has undergone an epidemiological shift, expanding its geographical footprint worldwide and causing huge economic impacts on the livestock industry. Vaccination is currently the only way to prevent the emergence, re-emergence, and incursion of LSD. However, due to limited research on the pathogenesis of LSDV, there are currently no safe and efficient vaccines for the prevention of LSD (1, 42). Given that viral strains lacking virulent genes can be used as potential vaccine candidates, we tried to identify key virulence genes associated with the imbalance of inflammatory response in LSDV-infected cattle and explore the underlying pathogenic mechanisms of these genes to provide targets for vaccine development.

Our results found that LSDV infection activated IL-1β and TNFα signaling and induced hyperinflammatory response. Through unbiased NF-κB luciferase reporter assays, we identified LSDV001 as a positive regulator of NF-κB activation. We found that overexpression of LSDV001 potentiated IL-1β-triggered activation of NF-κB, phosphorylation of TAK1 and IKK, and transcription of inflammatory cytokines in human HEK293 and bovine MDBK cells, whereas LSDV001-deficient virus had decreased ability to induce phosphorylation of TAK1, IκBα, and p65, as well as transcription of downstream antiviral genes. These results suggest that LSDV001 plays a key role in promoting NF-κB-dependent inflammatory response by targeting a component upstream of TAK1.

As a serine/threonine kinase, TAK1 is essential for IL-1β-triggered NF-κB activation. The activation of TAK1 depends on the assembly of the TAK1/TABs complex that induces auto- or para-phosphorylation of TAK1 (40). Our results indicated that LSDV001 interacted with TAK1 and TAB2/3. Domain mapping experiments showed that both the N-terminus and C-terminus of LSDV001 interacted with TAK1 and TAB2. The kinase domain of TAK1 and the CC-NZF domain of TAB2 were responsible for their association with LSDV001. Overexpression of LSDV001 promoted the assembly of the TAK1-TAB2/3 complex as well as its recruitment to TRAF6, an essential adaptor protein in the IL-1β-triggered signaling pathway. These results suggest that LSDV001 positively regulates IL-1β-triggered NF-κB activation and inflammatory response by promoting the recruitment of the TAK1/TABs complex to TRAF6, a process essential for IL-1β-triggered induction of downstream inflammatory genes.

Our results showed that LSDV001 markedly enhanced IL-1β-induced signaling in both human HEK293 and bovine MDBK cells, while LSDV001 weakly increased TNFα-induced signaling in bovine MDBK cells but had minimal effects in human HEK293 cells. The relatively weaker effects of LSDV001 on TNFα-triggered signaling may be attributed to the unique dual pathways responsible for TNFα-triggered NF-κB activation in these cells. In one process, TNFα binding to TNF-R1 induces the recruitment of the TAK1/TABs complex to TRAF2, leading to activation of TAK1 and subsequent IKK complex (43). Alternatively, TNFα binding to TNF-R1 causes polyubiquitination of RIP1, which recruits NEMO/IKKγ to directly activate the IKK complex (28). This alternative pathway potentially bypasses the need for the TAK1-TABs complex assembly, thereby reducing the contribution of LSDV001 to TNFα-triggered NF-κB activation and induction of downstream inflammatory genes. In addition, LSDV001 may have higher affinities to components of bovine TAK1-TABs complexes compared to their human orthologs, which may account for the observation that LSDV001 increases IL-1β-triggered signaling in bovine MDBK but has minimal effects in human HEK293 cells.

LSDV001 is classified as a BCL-2-like protein based on its predicted three-dimensional structure (44, 45). BCL-2-like proteins have been identified in three large DNA virus families infecting mammals: Herpesviridae, Adenoviridae, and Poxviridae. Although viral BCL-2 homologs share a conserved BCL-2-like fold, they differ substantially from one another and their cellular counterparts at both the sequence and functional levels (46). Some viral BCL-2-like proteins, such as vaccinia virus F1L and N1, myxoma virus M11L, parapoxvirus ORF virus ORFV125, and fowlpox virus FPV039 (47–51), exhibit classical anti-apoptotic functions similar to cellular BCL-2. These anti-apoptotic viral BCL2 proteins, through the conserved hydrophobic groove on their surface, bind to the BH3 peptides of pro-apoptotic BCL-2-like or BH3-only proteins to inhibit apoptosis.

During evolution, certain viral BCL-2-like proteins have lost the canonical function of anti-apoptosis due to occlusion of the BH3-binding groove, which renders them incapable of regulating apoptosis while retaining the overall BCL-2-like fold. Some of these proteins have evolved novel functions in modulating host innate immune responses, which are independent of the classical BCL-2 structural mechanisms (52, 53, 54). For example, VACV A52R mimics a truncated form of MyD88 and exerts dominant-negative effects on signaling triggered by IL-1, TLR3, and IL-18. A52R also activates p38 MAP kinase and enhances TLR4-induced production of IL-10 (55, 56). VACV OPG200 (also called B14) inhibits NF-κB-dependent gene expression by blocking the phosphorylation of IKKβ within the IKK complex (57). In our study, LSDV001 exhibits distinct functional and molecular properties compared to previously reported viral BCL-2-like proteins. Specifically, LSDV001 interacts with TAK1 and TAB2/3, promoting the assembly of the TAK1-TAB complex, thereby enhancing TNFα and IL-1β-mediated activation of NF-κB. These findings expand the known functional repertoire of viral BCL-2-like proteins in the regulation of host innate immune responses and provide new insights into the mechanisms by which structurally conserved viral proteins can acquire divergent immunomodulatory functions.

Our results showed that *LSDV001* was an early gene, and the deficiency of LSDV001 did not affect viral replication. However, LSDVΔ001 had attenuated ability to activate NF-κB, phosphorylation of TAK1 and IKK, and induce the expression of inflammatory cytokines compared with wild-type LSDV in MDBK cells. Consistent with the results *in vitro*, deficiency of LSDV001 reduced LSDV-induced secretions of TNFα and IL6 in C57BL/6 mice model. Syrian hamsters may be suitable laboratory models, developing skin nodules at the sites of inoculation upon LSDV infection (41). In our study, we observed the formation of nodules at the inoculation sites on the hamster skin, and infection with LSDVΔ001 led to smaller skin nodules and less inflammation than wild-type LSDV.

While many viral proteins have evolved mechanisms to suppress host immune responses for immune evasion, it is increasingly recognized that some viral virulence factors can promote disease severity by exacerbating inflammatory responses (58–60). In our study, LSDV001 promoted IL-1β- and TNFα-induced NF-κB activation, leading to increased expression of inflammatory cytokines. This proinflammatory effect contributes to the formation of nodular lesions and tissue damage, suggesting that LSDV001 functions as a virulence factor by exacerbating host inflammatory responses.

## MATERIALS AND METHODS

### Reagents, viruses, and cells

Fetal bovine serum (SA211.02, Cellmax); penicillin and streptomycin (SV30010, HyClone); Dulbecco’s modified Eagle’s medium (C11965500BT, Gibco); Puromycin (AMR-J593, VWR); Protein A + G agarose (P2055, Beyotime); PMSF (P7626, Sigma); recombinant human TNFα (10601, Sino Biological); recombinant human IL-1β (200-01B, PeproTech); recombinant bovine TNFα (2279-BT/C, R&D); Dual-Specific Luciferase Assay Kit (E1980, Promega); SYBR Green supermix (Q312, Vazyme); HiScript II Select RT SuperMix for qPCR (R323, Vazyme); ELISA MAX Deluxe Set Mouse TNFα and IL-6 (430904 and 431304, BioLegend); the inhibitors aprotin, leupeptin, β-glycerophosphate disodium salt, and sodium orthovanadate (HY-P0017, HY-18234A, HY-126304 and HY-D0852, MCE) were purchased from the indicated manufacturers.

Mouse monoclonal antibodies against HA (66006, Proteintech); rabbit monoclonal antibodies against HA (H6908, Sigma); Mouse monoclonal antibodies against Flag (F3165, Sigma); HRP-Flag (ZB15939, Servicebio); IgG (I5381 and I5006, Sigma); p-TAK1 (Thr184/187) (4508, Cell Signaling Technology), TAK1, TAB3, TRAF2, and TRAF6 (A19077, A19824, A19129, and A19686, ABclonal); TAB2 (3745, CST); β-actin and p65 (sc-47778 and 71675; Santa Cruz Biotechnology); p-p65(S536) and p-IKKα (Ser176)/IKKβ(Ser177) (3033 and 2078, Cell Signaling Technology); IκBα and p-IκBα (9242 and 9246, Cell Signaling Technology), anti-mouse IgG (H + L), F(ab′)2 Fragment (Alexa Fluor 594 Conjugate) (8890, Cell Signaling Technology); Alexa Fluor 488 goat anti-rabbit IgG (H + L) (A11008, Invitrogen) were purchased from the indicated manufacturers. The LSDV001 antibody was commissioned for preparation by GenScript.

The LSDV strain used in this study, designated LSDV/China/Hainan/2021, was kindly provided by Mr. Huaijie Jia of Lanzhou Veterinary Research Institute. This strain was isolated from clinically infected cattle during a lumpy skin disease outbreak in Hainan Province, China, in 2021. Whole-genome sequencing was performed, and the complete genome sequence has been submitted to GenBank (accession: PV796583). Sequence alignment indicated >99.5% identity with the Neethling-type field strain (GenBank accession: AF409138), confirming that LSDV/China/Hainan/2021 is a wild-type isolate.

HEK293 (CRL-1573) and HeLa (CCL-2) cells were obtained from the American Type Culture Collection (ATCC). MDBK cells (GDC0108) were purchased from the China Center for Type Culture Collection (CCTCC).

### Plasmids

The LSDV protein expression clones were synthesized by GenScript. Expression plasmids for Flag-tagged LSDV001, HA-tagged TAK1 and its truncation mutants, TAB1, TAB2 and its truncation mutants, and TAB3 and IKKβ were constructed by standard molecular biology techniques.

### Generation of stable cell lines

Double-stranded oligonucleotides corresponding to the *LSDV001* gene sequence were cloned into the pCDH vector, which was co-transfected with the packaging plasmids psPAX2 and pCMV-VSV-G into HEK293 cells. Forty-eight hours after transfection, the viruses were harvested for infection of MDBK cells. The infected cells were selected with puromycin (1.5 µg/mL) for 6 days to establish stable cell lines.

### Generation of LSDV001 and LSDV156-deficient virus (LSDVΔ001/Δ156)

LSDVΔ001/Δ156 was generated by homologous recombination in MDBK cells. The recombinant plasmid was first constructed using pUC18 as the backbone. The recombination cassette contained left and right homology arms flanking the *LSDV001* and *LSDV156* locus, as well as the *EGFP* reporter gene driven by the LSDV pA7L promoter, which was inserted into the coding region of *LSDV001* and *LSDV156*. MDBK cells were infected with wild-type LSDV for 12 hours and then transfected with the homologous recombinant plasmid using Fugene transfection reagent. After culturing for 48 hours, the cells were frozen-thawed and seeded with MDBK cells in 96-well plates by limiting dilution assay. After several rounds of dilution screening and amplification, the purified LSDVΔ001 was obtained. The purity of LSDVΔ001 was determined by PCR and immunoblot analysis. The sequences of the PCR primers were as follows:

Primer pair 1: 5′-AATGCCAATCACTGCACATG-3′ and

5′-ATGTCTTCCGGCAACTATGT-3′;

Primer pair 2: 5′-AATGCCAATCACTGCACATG-3′ and

5′-ACGCTGAACTTGTGGCCGTT-3′.

### qPCR

Total RNAs were isolated, and the reverse transcribed products were obtained for qPCR analysis to measure mRNA levels of the indicated genes. The data shown were the relative abundance of the indicated mRNA normalized to that of *GAPDH*. Total DNA was extracted using a commercial viral DNA extraction kit (DP315, TIANGEN). The sequences of the qPCR primers were as follows:

Human *GAPDH*: 5′-GACAAGCTTCCCGTTCTCAG-3′ and

5′-GAGTCAACGGATTTGGTCGT-3′;

Human *TNFA*: 5′-GCCGCATCGCCGTCTCCTAC-3′ and

5′-CCTCAGCCCCCTCTGGGGTC-3′;

Human *IL8*: 5′-GAGAGTGATTGAGAGTGGACCAC-3′ and

5′-CACAACCCTCTGCACCCAGTTT-3′;

Human *CXCL10*: 5′-GGTGAGAAGAGATGTCTGAATCC-3′ and

5′-GTCCATCCTTGGAAGCACTGCA-3′;

Human *IΚΒΑ*: 5′-CGGGCTGAAGAAGGAGCGGC-3′ and

5′-ACGAGTCCCCGTCCTCGGTG-3′;

Bovine *GAPDH*: 5′-AGGTCGGAGTGAACGGATTC-3′ and

5′-ATGGCGACGATGTCCACTTT-3′;

Bovine *IL1B*: 5′-AGTGCCTACGCACATGTCTTC-3′ and

5′-TGCGTCACACAGAAACTCGTC-3′;

Bovine *IL8*: 5′-CCAATGGAAACGAGGTCTGC-3′ and

5′-TTGCTTCTCAGCTCTCTTCAC-3′;

Bovine *IL6*: 5′-AACCACTCCAGCCACAAACACT-3′ and

5′-CTCAGGCTGAACTGCAGGAA-3′;

Bovine *CXCL10*: 5′-CTCGAACACGGAAAGAGGCA-3′ and

5′-TCCACGGACAATTAGGGCTT-3′;

Bovine *TNFA*: 5′-GTCTGCCATCAAGAGCCCTT-3′ and

5′-ACTGAGGCGATCTCCCTTCT-3′;

Bovine *CCL2*: 5′-TGCAGACCCCAAGCAGAAAT-3′ and

5′-AGAGGGCAGTTAGGGAAAGC-3′;

*LSDV001*: 5′-TGGCATTTTCCTCCAGGGAG-3′ and

5′-AATGCCAATCACTGCACATG-3′;

*LSDV031*: 5′-ACAGTTGAATGTGATGGCGA-3′ and

5′-TGGGGATGAAGCTCTTGCAG-3′;

*LSDV063*: 5′-TGTTCATTCACCATCCGCATC-3′ and

5′-GGTTCTTGTAATGGCTTGTTGC-3′.

### Transfection and reporter assays

HEK293 cells were transfected with the indicated plasmids by the calcium phosphate precipitation method. To normalize for transfection efficiency, pRL-TK (Renilla luciferase) reporter plasmid (20 ng) was added to each transfection. 24 hours after transfection, cells were treated or left untreated with the indicated stimuli before luciferase assays were performed using a dual-specific luciferase assay kit. Firefly luciferase activities were normalized with Renilla luciferase activities.

### Confocal microscopy

HeLa cells were seeded on coverslips in 24-well plates and transfected with the indicated plasmids for 24 hours. The cells were fixed with 4% paraformaldehyde for 20 min and then permeabilized for 15 min by incubation with 0.1% Triton X-100. Subsequently, the cells were blocked in 1% BSA and stained with the indicated antibodies. Imaging of the cells was carried out using a Zeiss confocal microscope.

### Coimmunoprecipitation and immunoblot analysis

Cells were lysed with lysis buffer (20 mM Tris-HCl, pH 7.5; 1% Nonidet P-40; 10 mM NaCl; 3 mM EDTA and 3 mM EGTA, complete protease inhibitor mixture) at 4°C for 10 min and sonicated. The lysates were centrifuged at 13,000 rpm for 10 min at 4°C. The supernatants were immunoprecipitated with the indicated antibodies and protein A + G agarose and then incubated at 4°C for 3 hours. Then the beads were washed four times with washing buffer (750 mM NaCl, 50 mM Tris-HCl, pH 7.5). The bound proteins were separated by SDS-PAGE, followed by immunoblotting analysis with the indicated antibodies.

### Viral plaque assay

MDBK cells were infected with wild-type LSDV or LSDVΔ001 (MOI = 0.01) for the indicated times. The cells and supernatants were harvested and freeze-thawed to obtain the suspensions. MDBK cells were seeded in 24-well plates, and the cells were infected by incubation with serial dilutions of the suspensions for 2 hours at 37°C. The infected cells were overlaid with 1.5% methylcellulose and were then incubated for 96 hours before plaque counting.

### Animal experiments

Six-month-old Holstein cattle were purchased from Gansu Qianjin Animal Husbandry Technology Co., Ltd. Cattle were intravenously injected with wild-type LSDV (2 × 10^6^ pfu) for 14 days, and then lesion (L) and non-lesion (N) skin samples from the same cattle were dissected for qPCR experiments.

Seven-week-old C57BL/6 mice were purchased from Lanzhou Veterinary Research Institute of the Chinese Academy of Agricultural Sciences. Mice were intravenously injected with wild-type LSDV or LSDVΔ001 (1 × 10^6^ pfu) for 24 hours, followed by orbital blood collection for ELISA.

Three-week-old LVG hamsters were purchased from Beijing Vital River Laboratory Animal Technology Co., Ltd. Hamsters were intradermally injected with wild-type LSDV or LSDVΔ001 (5 × 10^5^ pfu) for 5 days, and then nodular skin lesions were fixed with 4% paraformaldehyde immediately. Paraffin-embedded sections of the skins were subjected to HE staining or DAPI staining and then examined by microscopy.

### Facility biosafety statement

All experiments with live LSDV were conducted in the National Foot and Mouth Disease Reference Laboratory (ABSL-3) at the Lanzhou Veterinary Research Institute of the Chinese Academy of Agricultural Sciences. The experiments were approved by the Ministry of Agriculture and Rural Affairs (approval number: 07140020250302) and the China National Accreditation Service for Conformity Assessment (approval number: CNAS BL0098).

### Statistics analysis

Unpaired Student’s *t* test was used for statistical analysis with GraphPad Prism Software. The number of asterisks represents the degree of significance with respect to *P* values. Statistical significance was set at *P* < 0.05, * or *P* < 0.01, **.
